# Analysis of the Prevalence of HTLV-1 Proviral DNA in Cervical Smears and Carcinomas from HIV Positive and Negative Kenyan Women

**DOI:** 10.3390/v8090245

**Published:** 2016-09-05

**Authors:** Xiaotong He, Innocent O. Maranga, Anthony W. Oliver, Peter Gichangi, Lynne Hampson, Ian N. Hampson

**Affiliations:** 1 Viral Oncology Lab, University of Manchester, St Mary’s Hospital, Manchester M13 9WL, UK; xiaotong.he@manchester.ac.uk (X.H.); dr.maranga@yahoo.com (I.O.M.); Anthony.W.Oliver@manchester.ac.uk (A.W.O.); lynne.hampson@manchester.ac.uk (L.H.); 2 Obstetrics and Gynaecology, University of Nairobi, Kenyatta National Hospital Nairobi, Nairobi 00202, Kenya; gichangip@yahoo.com

**Keywords:** human immunodeficiency virus (HIV), human T-cell lymphotropic virus type 1 (HTLV-1), human papilloma virus (HPV), retrovirus, liquid based cytology (LBC), invasive cervical cancer (ICC), proviral DNA, PCR

## Abstract

The oncogenic retrovirus human T-cell lymphotropic virus type 1 (HTLV-1) is endemic in some countries although its prevalence and relationship with other sexually transmitted infections in Sub-Saharan Africa is largely unknown. A novel endpoint PCR method was used to analyse the prevalence of HTLV-1 proviral DNA in genomic DNA extracted from liquid based cytology (LBC) cervical smears and invasive cervical carcinomas (ICCs) obtained from human immunodeficiency virus-positive (HIV+ve) and HIV-negative (HIV−ve) Kenyan women. Patient sociodemographic details were recorded by structured questionnaire and these data analysed with respect to HIV status, human papillomavirus (HPV) type (Papilocheck^®^) and cytology. This showed 22/113 (19.5%) of LBC’s from HIV+ve patients were positive for HTLV-1 compared to 4/111 (3.6%) of those from HIV−ve women (*p* = 0.0002; odds ratio (OR) = 6.42 (2.07–26.56)). Only 1/37 (2.7%) of HIV+ve and none of the 44 HIV−ve ICC samples were positive for HTLV-1. There was also a significant correlation between HTLV-1 infection, numbers of sexual partners (*p* < 0.05) and smoking (*p* < 0.01). Using this unique method, these data suggest an unexpectedly high prevalence of HTLV-1 DNA in HIV+ve women in this geographical location. However, the low level of HTLV-1 detected in HIV+ve ICC samples was unexpected and the reasons for this are unclear.

## 1. Introduction

Human T-cell lymphotropic virus type 1 (HTLV-1) was the first pathogenic human retrovirus to be discovered and is known to be associated with various mild to severe pathologies resulting from chronic lifelong infection [1,2,3,4]. It is the aetiological agent for adult-T-cell leukaemia (ATL) and HTLV-1-associated myelopathy/tropical spastic paraparesis (HAM/TSP) with the former often proving fatal whilst the latter causes significant debilitating morbidity. Other associated diseases include arthropathy, HTLV-1 associated infective dermatitis, uveitis and polymyositis [5,6,7]. The cumulative risk of a HTLV-1 carrier developing ATL has been estimated at between 2.5% and 5% although a latency period of 50–70 years is typical [8,9].

Although HTLV-1 is reported to currently infect between 10 and 25 million people globally, the true prevalence in different geographical regions of the world is largely unknown [4,7,10,11]. The 2012 review of global HTLV-1 prevalence by Gessain and Cassar [11] summarises the most recent data and contains comprehensive details of patient numbers, subgroups studied and outcomes. Current estimates rely on serological screening of blood donors and pregnant women which often excludes high-risk groups such as human immunodeficiency virus (HIV) positive individuals and ethnic minorities [4,7]. HTLV-1 infection is endemic in many parts of the world including southern Japan, Equatorial Africa, Central and South America and in immigrant descendants of people from these regions [12]. In a recent review of global HTLV-1 prevalence, the highest prevalence was found in the Japanese islands with 36.4%. Other reports in Africa are between 6.6% and 8.5% in Gabon and 1.05% in Guinea whereas elsewhere, such as in the Caribbean islands, it is 6% [4,7,13]. The prevalence in female sex workers has ranged from 3.2% in Congo, to 5.7% in Fukuoka (Japan) and from 8.7% to 21.8% in Callao (Peru) [14]. A study done in São Paulo (Brazil) on HIV positive intravenous drug addicts (IVDAs) showed a HTLV-1 prevalence of 15.3% [15].

Both HIV and HTLV-1 share similar risk factors since they are both transmitted through sexual contact, blood transfusion, sharing of needles among intravenous drug users and vertical transmission from mother to child via breast-feeding, [8,10,16,17]. With regard to breast-feeding, transmission of HTLV-1 via this modality is known to be related to duration, proviral load and antibody titre in addition to other less well defined factors [18]. The development of neoplasia in patients with HIV and acquired immune deficiency syndrome (HIV/AIDS) is generally attributed to failure of immune surveillance and associated co-infections with oncogenic viruses such as human herpes virus 8 (HHV8) and Kaposi sarcoma, Epstein–Barr virus (EBV) with non-Hodgkins lymphoma (NHL), and human papilloma virus (HPV) with invasive cervical carcinomas (ICC) [19,20,21,22].

During the past 20 years, HIV type 1 (HIV-1)/HTLV-1 co-infection has emerged as a global health problem with increasing numbers of cases in South America and Africa [8]. Although both viruses have a tropism for CD4+ T helper cells, the overall effect of virus-virus interactions on their related pathologies is still controversial [8]. Although Africa is considered to be a large reservoir for HTLV-1 infection, there is a paucity of prevalence data from Sub-Saharan Africa and especially East Africa [7,11]. Based on screening pregnant women, in Western Africa Nigeria has been reported to have the highest prevalence of 5.5% followed by Zaire in Central Africa at 4.6% where subtype B is the most common [11]. It is thus significant that screening for HTLV-1 during pregnancy and before blood transfusion is not routine in most Sub-Saharan African countries [10,23]. Furthermore, it is also very likely that other higher-risk groups, such as intravenous drug addicts (IVDA) and sex workers, will have a much higher prevalence of the virus. As mentioned the study of IVDA’s in São Paulo found 15.3% prevalence [15] which is much higher than the 0.1% prevalence observed in pregnant women in the same location [11]. Since there is, as yet, neither an effective vaccine against HTLV-1 nor a cure for its associated pathologies, the virus has the potential to impose a significant social and financial burden [10,24] in this area of the world. Moreover, given that Sub-Saharan Africa is also home to two thirds of the global HIV pandemic, it is important to assess how these two retroviruses associate in this population and how this may contribute to the pathology of different diseases.

Several previous studies have attempted to link HTLV-1 infection with the aetiology of ICC and have produced inconsistent findings. For example, a study carried out in Yucatan (Mexico) where HTLV-1 prevalence is low but the incidence of ICC is high, found no statistical difference between these two groups [25]. However, a larger study carried out in Japan found the prevalence to be higher in ICC patients younger than 59 years. Furthermore, increased numbers of HTLV-1 infections were also found in women from all age groups who developed vaginal carcinomas (VC) when compared to age-matched healthy controls [26]. The conclusion from this study was that HTLV-1 infection may promote cervical carcinogenesis and may also affect the prognosis of ICC or VC. These results were consistent with a study carried out on Jamaican women where a higher prevalence of HTLV-1 was found in cervical intraepithelial neoplasia 3/invasive cervical cancer (CIN3/ICC) patients when compared to controls [27]. However, these data were not supported in a more recent report [28].

In light of these previous observations, the objective of the current study was to evaluate the prevalence of HTLV-1 proviral DNA extracted from liquid based cytology (LBC) specimens from HIV positive and negative patients attending specialist referral clinics in Kenyatta National Hospital in Nairobi, Kenya. The same analysis was carried out on DNA extracted from biopsies obtained from patients with ICC in the same hospital.

## 2. Materials and Methods

### 2.1. Study Population and Sample Collection

A cross-sectional study was carried out among women attending Kenyatta National Hospital (KNH), Nairobi, Kenya. Consecutive female patients attending a Specialist HIV Clinic and Family Planning Clinic were recruited between April 2008 and February 2009. Women aged between 21 and 52 years were included, while those who had prior destructive procedures for cervical disease and/or hysterectomy were excluded. A total of 224 patients were recruited, including 111 HIV negative (median age = 35 years, range = 21–52) and 113 HIV positive (median age = 35 years, range = 21–52) with 56 of these in receipt of highly active antiretroviral therapy (HAART). After undergoing voluntary counselling and testing for HIV, a structured questionnaire was administered and a blood sample was collected. During the same period, 37 HIV positive and 40 HIV negative women newly diagnosed with ICC at KNH were also randomly recruited. They underwent examination under anaesthesia (EUA), a biopsy was taken which was then formalin-fixed and paraffin-embedded (FFPE). FFPE samples were used for histopathology and DNA extractions.

This study was approved by the Kenyatta National Hospital’s Ethics and Research Board (ERB), the University of Nairobi (No KNH-ERC/01/4988) and the University of Manchester, Oldham Research Ethics Committee amendment 5 project 07/Q1405/14. All patients gave written informed consent.

### 2.2. LBC Samples

All patients were examined with a speculum and cervical samples collected using a cervex brush, which was stirred into a vial of PreservCyt® transport solution (ThinPrep® Pap test, Hologic Inc., Bedford, MA, USA). Cytology slides were prepared using a Cellspin Cytocentrifuge, (Tharmac, Waldsolms, Germany) and were stained with Papanicolaou (pap) stain. All pap stained slides were independently examined by two different pathologists and the Bethesda 2001 criteria were used for slide interpretation [29].

### 2.3. HIV Testing

HIV testing was done using Determine^®^ test kit (Abbot Pharmaceuticals, Chicago, IL, USA), and, if positive, this was confirmed by Uni-Gold^®^ (Trinity Biotech Plc, Bray, Ireland).

### 2.4. Extraction of Genomic DNA

All residual PreserveCyt® material was used for automated DNA extraction using BioRobot^®^ M48 (Qiagen, Hilden, Germany) as described by the manufacturer. Approximately 4 × 10 µm FFPE ICC sections were used for DNA extraction using the Qiagen Qiacube® (Qiagen, Crawley, West Sussex, UK) according to the manufacturer’s instructions.

### 2.5. Papillocheck^®^ HPV Genotyping

DNA was quantified using a Nanodrop UV spectrophotometer (Thermo Fisher, Altrincham, Cheshire, UK) and used for HPV genotyping with the PapilloCheck^®^ test (Greiner Bio-One, Stonehouse, Gloucestershire, UK) as per the manufacturer’s instructions. This identifies 24 different HPV genotypes: six low-risk HPV types (LR: 6, 11, 40, 42, 43, and 44/55) and 18 high-risk HPV types (HR: 16, 18, 31, 33, 35, 39, 45, 51, 52, 53, 56, 58, 59, 66, 68, 70, 73, and 82).

### 2.6. PCR

GAPDH PCR was performed to assess the quality of input DNA using a 50 µL reaction mixture containing 2 µL of each DNA sample (50 ng), 2.5 units of BioTaq DNA polymerase (Bioline Ltd., London, UK), 0.2 mM dNTPs, and 0.2 µM of each primer in 10 mM Tris-HCl pH 8.3, 50 mM KCl and 2.5 mM MgCl_2_. HTLV-1 Tax PCRs were optimized using a multiplex PCR kit (Qiagen) as recommended by the manufacturer. All reactions were carried out in duplicate, using a Veriti™ Thermal Cycler (Applied Biosystems, Paisley, UK) with the conditions and primers indicated in Table 1. The sensitivity of the method was tested by adding a dilution series of genomic DNA extracted from Tax transduced JPX-9 cells [30] to 50 ng of genomic DNA from a HTLV-1 negative patient per reaction. PCR products were separated by 1.5%–2.5% agarose gel electrophoresis, stained with ethidium bromide and examined under UV.

### 2.7. DNA Sequencing

The gel-separated DNA bands were excised, isolated, purified and sequenced using an ABI BigDye Cycle sequencing kit (Applied Biosystems, Warrington, UK) as indicated by the manufacturer and a 3100 ABI sequencer (Applied Biosystems, Warrington, UK).

### 2.8. Statistical Analysis

The data were captured in an Access database and exported to SPSS version 16.0 for analysis after cleaning and validation. Statistical tests of significance were done using Pearson’s chi-square with Yates correction or with Fisher’s test if the expected frequencies were less than five. Odds ratio (OR), adjusted OR (AOR) and the 95% confidence intervals (CI) were used to measure strengths of associations. Further, cross-tabulations were also used to assess the distribution between HTLV-1 and HIV status of the participants, and the 95% CI around the prevalence of virus infection was computed using R program version 3.2.2 package binom. A *p*-value of less than 0.05 was considered statistically significant.

## 3. Results

### 3.1. DNA Quality

As indicated, PCR of glyceraldehyde 3-phosphate dehydrogenase (GAPDH) with GAPDH specific primers was used to assess the quality and integrity of all DNA samples prior to these being used for unknown test PCR’s with Tax specific primers as illustrated in Figures S1 and S2.

### 3.2. PCR Detection of Proviral HTLV-1 Tax DNA

**Figure 1:**
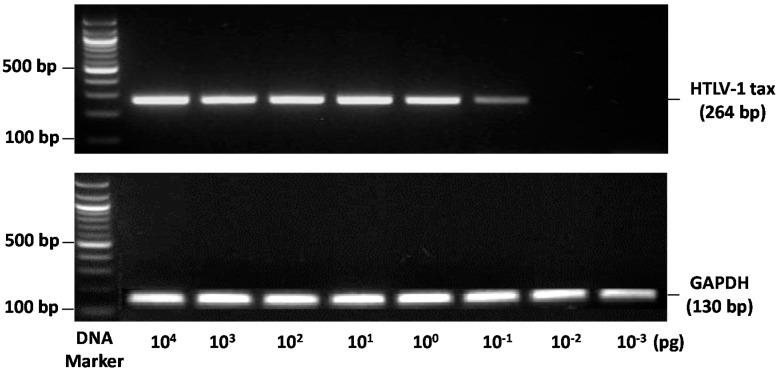
Sensitivity of human T-cell lymphotropic virus type 1 (HTLV-1) Tax PCR detection. End-point PCR method able to specifically detect a single HTLV-1 Tax DNA amplimer from 0.1 fg of input genomic DNA (DNA from the human JPX9 cell line was used as a positive control for Tax). GAPDH: glyceraldehyde 3-phosphate dehydrogenase.

As illustrated in Figure 1, this simple hot-start, end-point PCR method specifically detected a single 264 bp HTLV-1 Tax DNA amplimer from as little as 0.1 fg of input genomic DNA from the JPX-9 cell line.

The specificity of the single Tax amplimer product was confirmed by excision and DNA sequencing of the 264-bp PCR product and the identity verified by the use of National Center for Biotechnology Information (NCBI) Basic Local Alignment Search Tool (BLAST) (See Supplementary Data S1, Figure S4 and Supplementary Data S2 (BLAST)).

### 3.3. Analysis of the Association between HTLV-1 Infection and HIV Infection in LBC DNA

Genomic DNA extracted from 113 HIV positive and 111 HIV negative LBC samples was analysed for the presence of HTLV-1 Tax proviral DNA. As shown in Figure S1, a single HTLV-1 Tax amplimer was successfully amplified from 26 out of 224 of the LBC samples giving a total infection rate of 11.6% (95% confidence interval (CI): 7.7%–16.5%). Most significantly, it was found that 22 of the HTLV-1 positive samples were also HIV positive producing an infection rate of 19.5% (95% CI: 12.6%–27.9%) in the 113 HIV positive patients.

The HTLV-1 prevalence was four out of 111 (3.6%) (95% CI: 0.9%–8.9%) in HIV negative women, which indicates that the frequency of HTLV-1 infection is >5 times higher in HIV positive than negative patients, indicating that HTLV-1 infection is significantly associated with a HIV positive status (*p* < 0.01).

### 3.4. Analysis of the Association of HTLV-1 Infection with Abnormal Cervical Cytology

Using the same LBC samples, we have previously shown that HIV infection was associated with abnormal cervical cytology [31]. In light of this, HTLV-1 infection was assessed in relation to our previous cervical cytology findings where low-grade and high-grade squamous intraepithelial lesion (LSIL and HSIL) and abnormal squamous cells of undetermined significance (ASCUS) were analysed independently. In contrast to HIV infection, there was no statistically significant evidence for an association between HTLV-1 and abnormal cervical cytology (*p* = 0.231 using Pearson’s chi-square with Yates correction). Nevertheless, it was noted that all the HTLV-1 infections were in normal/LSIL and none were found in HSIL/ASCUS (See Figure S4).

### 3.5. Analysis of the Association between HTLV-1 Infection, Stage of HIV and Use of Highly Active Antiretroviral Therapy (HAART)

According to World Health Organization (WHO) clinical staging system, the 113 HIV positives were classified as stage I–IV, consisting of stage I in 24, II in 16, III in 38 and IV in 35 patients. There was no statistical significance between HTLV-1 positive and HIV clinical stages. Additionally, HTLV-1 positivity was not associated with increasing advancement of HIV disease or use of HAART (*p* > 0.05) (See Table S3). WHO guidelines for HIV staging can be found on the AIDS Education and Training Center (AETC) website [32].

### 3.6. Analysis of the Association between HTLV-1 Infection and HPV in LBC DNAs

**Figure 2:**
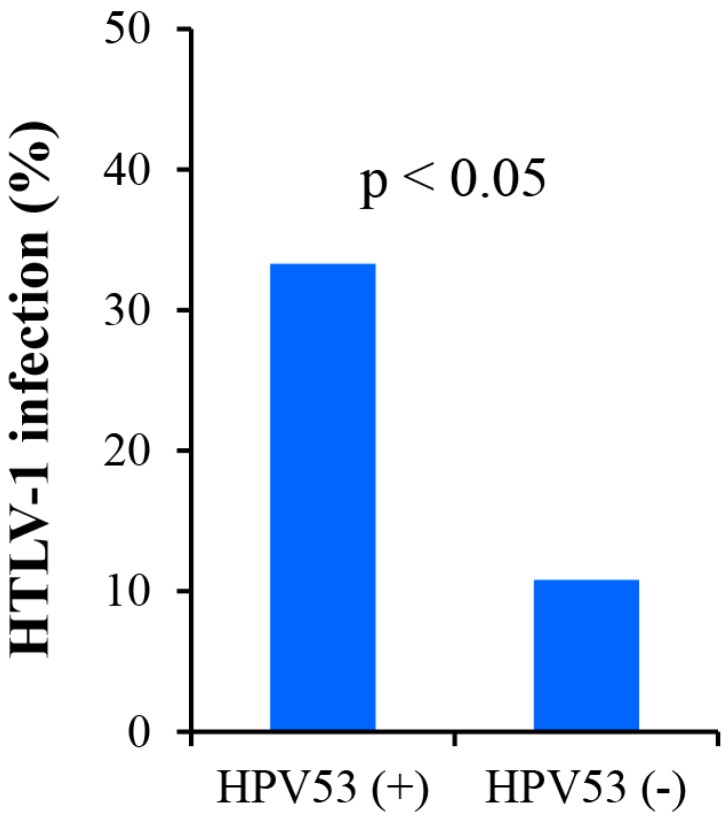
Association of HTLV-1 and human papillomavirus (HPV) type 53 infections. HPV type 53 showed a statistically significant association with HTLV-1 infection (p <0.05). None of the other HPV sub-types showed a positive association.

As described above, 24 subtypes of HPV, including six low-risk (LR) and 18 high-risk (HR) were identified in LBC specimens using the Papillocheck^®^ system. Of the samples analysed, one sample had an invalid result, while 121 (55%, 95%CI: 48.2%–61.7%) had at least one HPV subtype with the highest number of genotypes detected in any one individual being seven, covering 107 HR (88.4%, 95% CI: 81.3%–93.5%) and 14 LR (11.6%, 95% CI: 6.5%–18.7%) HPV subtypes. None of the HPV subtypes covered showed any association with HTLV-1 apart from HPV type 53 where there was a significant positive association (*p* < 0.05) (Figure 2 and Table 2).

### 3.7. Analysis of the Relationship between HTLV-1 Infection and Patient Age

**Figure 3:**
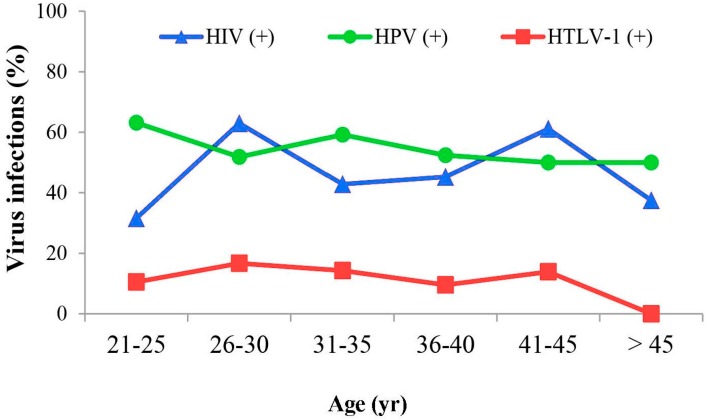
Relationship between HTLV-1, HPV, human immunodeficiency virus (HIV) infections and age. There was no significant difference observed between the median age of HTLV-1 positives with respect to either HPV or HIV status. However, there was a trend towards a lower HTLV-1 prevalence among women >45 years old when compared to women positive for HPV and HIV.

LBC material for this study was obtained from women with an age range of 21 to 52 years and median or mean ages of 35 or 35.2 years, respectively. The median age of HTLV-1 negatives was 35 and HTLV-1 positives 31 years with no significant difference observed between the median age of HTLV-1 positives with respect to either HPV or HIV status (Figure 3). Within HTLV-1 positives, no significant difference was found between infection and any of the age groups.

Among the 77 ICC study subjects, the median and mean ages were 42 and 44 years, respectively, with a range of 27–68 years. Any association between HTLV-1 infection and age could not be calculated since only one ICC sample was positive.

### 3.8. Analysis of the Relationship between HTLV-1 Infection and Patient Sociodemographics

**Figure 4:**
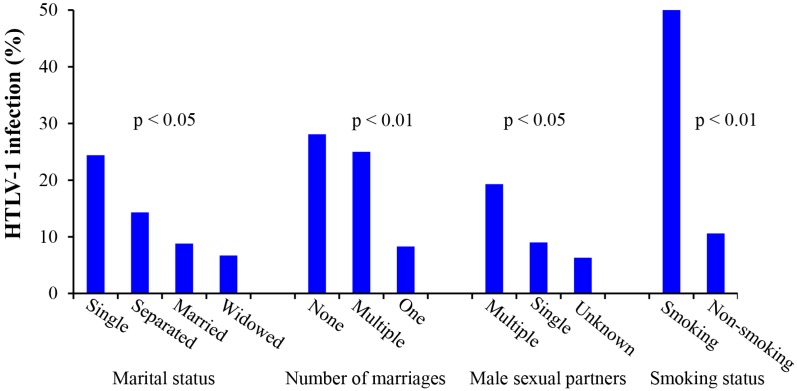
Analysis of the relationship between HTLV-1 infection and life styles factors. The rate of HTLV-1 infection in the smoking group was five times higher than that in non-smoking group. Women with increased numbers of marriages and sexual partners were significantly more likely to have HTLV-1 infections confirming that sexual contact is an important means of HTLV-1 transmission.

Analysis of the relationship between HTLV-1 infection and patient’s life style shown in Figure 4 indicated that the rate of HTLV-1 infection in the smoking group was five times higher than that in the non-smoking group. Women with increased numbers of marriages and sex partners were also significantly more likely to have HTLV-1 infections confirming that sexual transmission is an important means of HTLV-1 infection. No significant associations between HTLV-1 infections were found with religion, educational level, occupation, socio-economic status, contraceptive use, intravenous drug use, blood transfusion, genital herpes and either rural or urban residency (see Figure S5).

### 3.9. Analysis of HTLV-1 DNA in ICC DNA’s

HTLV-1 DNA was only detected in one out of 77 ICC biopsies, of which 37 were HIV positive (see Figure S2). This equates to an overall infection rate of 1.3% (95% CI: 0.033%–7%), which is significantly lower than the 12.1% (95% CI: 8.1%–17%) found in DNA from LBC material. However, if the HTLV-1 infections found in LBC and ICC material from just HIV positive women are compared, this equates to 19.5% (95% CI: 12.6%–27.9%) versus 2.7% (95% CI: 0.068%–14%), respectively. Thus, HIV positive women with ICCs have approximately seven-fold less numbers of HTLV-1 infections than would be predicted from the rate observed in LBC material from HIV positive women.

## 4. Discussion

Although HTLV-1 was discovered 35 years ago, information on its prevalence and associated risk factors in Sub-Saharan Africa countries have remained poorly defined. HIV, HTLV-1 and HPV can share the same sexual route of transmission and, to our knowledge, this is the first study carried out in Kenya to analyse the prevalence of HTLV-1 in LBC cervical specimens and formalin fixed and paraffin-embedded (FFPE) ICC biopsies from HIV positive and negative women. The most significant observation was the high prevalence of HTLV-1 proviral DNA found in LBC samples from HIV positive women, consistent with previous work carried out in Guinea-Bissau, showing that HIV infection is a potential risk factor for acquiring HTLV-1 [33]. Moreover, the seven-fold lower incidence of HTLV-1 DNA found in ICCs from HIV positive women begs the question, what is the explanation for this? Clearly, it could be related to differences in the type of sample and extraction procedures used for LBC’s and ICCs although there are other possibilities. For example, women who are positive for both HTLV-1 and HIV have increased AIDS related mortality, which could prevent them from living long enough to develop ICC. However, it is still not established that concomitant HTLV-1/HIV infection does promote progression to AIDS [8,34].

A limitation of the current study is the lack of corroborating serological data on HTLV-1 prevalence. Although the PCR method is sensitive, it is very clear this may underestimate the true prevalence since it depends on the inclusion of sufficient numbers of infected cells in extracted material. Indeed, this could also provide an alternative explanation for the lower detection of HTLV-1 DNA found in ICC samples. However, another possibility is that HTLV-1 could suppress HPV mediated cervical carcinogenesis in HIV positive women, which raises the question: is there a potential mechanism for such an effect? Both HIV and HTLV-1 infect CD4+ cells and it has been previously shown that elevated numbers of CD4+ cells are associated with HPV-related dysplasia [35,36,37]. Moreover, persistent HPV infections are known to suppress T-helper type 1 (Th-1) and promote a T-helper type 2 (Th-2) responses, which results in defective interferon (IFN) production. This, in turn promotes the development of HPV related neoplasia [38]. Thus, if co-infection with HTLV-1 and HIV produced a shift from Th-2 to a Th-1 response, this could potentially inhibit cervical carcinogenesis and explain the reduced prevalence of HTLV-1 in ICCs from HIV positive women. Indeed, it is highly significant that the work of Abrahäo et al. has shown that concomitant HTLV-1/HIV infection augments production of the Th-1 cytokines interleukin-2 (IL-2) and IFN above that of single HTLV-1 or HIV infected individuals which is entirely consistent with this hypothesis [39]. It is also curious that none of the 22 HPV subtypes tested, apart from HPV 53, showed any association with HTLV-1. The importance of this is that, in an earlier report, we demonstrated that type 53 was significantly more common in LBC’s from HIV positive than HIV negative women although it was never detected in any of the ICCs examined [31].

With regard to other cancers, a protective role for HTLV-1 is not without precedent. Hirata et al. evaluated the relationship between HTLV-1 infection and the occurrence of several types of cancers namely biliary tract, pancreatic, esophageal, gastric, colorectal, liver, and lung cancers using logistic regression analysis adjusted for age and sex [40]. Curiously, the HTLV-1 infection rate found in gastric cancer patients was significantly lower than in controls (*p* = 0.01) but no associations were observed with any of the other cancers studied.

Another important question raised by the current study is that, given the high prevalence of HTLV-1 in HIV positive Kenyan women, is there a correspondingly higher incidence of HTLV-1 related pathologies in these women as observed in Japan [41]? However, this may not be apparent as the average 59 years life expectancy in Kenya is much shorter than the 83 years in Japan. Since HTLV-1 related adult-T-cell leukaemia occurs after a long latency period, typically spanning 5–7 decades, it is thus highly likely that infected individuals in Sub-Saharan Africa will succumb to other illnesses prior to the development of HTLV-1 related pathologies. Indeed the median age for LBC and ICC subjects in the current study was 35 and 42 years, respectively.

Other noteworthy finding from our study was the significant association between HTLV-1 infection and increased numbers of direct and indirect sexual contacts in addition to smoking. Given that smoking is known to impair the function of cervical Langerhans cells, it is possible that the increased incidence of HTLV-1 in smokers could be related to impaired local immunity which contributes to increased rates of sexual transmission of the virus [42].

In summary, it is known that the influence of HIV infection on the incidence of HPV related ICC seems to vary depending on the geographical location of the study [43]. For example, work carried out in European locations shows an increased incidence of ICC in HIV positive women [43,44,45], whereas this has not been observed in other, mainly African, studies [46,47]. In light of our findings, it is tempting to speculate that the observed differences in ICC incidence rates found in HIV positive women in different geographical locations may be, at least in part, due to variations in the prevalence of HTLV-1 infection since this is much more common in Sub-Saharan Africa than Europe. Clearly, larger studies are needed in order to improve the statistical significance of this finding, which could also be supplemented with analysis of Th-1 and Th-2 cytokine profiles.

As discussed, there is a paucity of epidemiological data on HTLV-1 in Sub-Saharan Africa, which also bears the heaviest global HIV burden. The HTLV-1 prevalence found in LBC samples from HIV positive women (20.4%) suggests this could be higher than anticipated reinforcing the finding that Kenya may be endemic for HTLV-1 and also confirming the association between HTLV-1 and HIV. Clearly, there is an urgent need to conduct larger population based studies and to institute public health measures to curb the continued spread of HTLV-1. For example, addressing the issue of vertical transmission through breast-feeding (by considering safe replacement feeds for those whose mothers are carriers), increased condom use to curb sexual transmission and provision of universal HTLV-1 screening on all blood donations, tissue and organ donors. Currently, routine HTLV-1 screening is not carried out in most African countries including Kenya—a gap which may be promoting the spread of this virus. Furthermore, there is no curative treatment for HTLV-1 or its associated pathologies and an effective vaccine is equally unavailable which puts a heavy social and financial burden on sufferers, their families and the healthcare systems. Thus, public health interventions aimed at counselling and educating high-risk individuals and the public are of critical significance [10].

## Figures and Tables

**Table 1:** Primers and PCR conditions used.

Primer	Sequence	Conditions	Amplimer Size (bp)
GAPDH-F	5‘-CATTGACCTCAACTACATGGT-3‘	94 °C × 5 min; 33 cycles: 94°C × 25 s; 53 °C × 25 s; 72 °C × 25 s; 72°C × 7 min	130
GAPDH-R	5’-TCGCTCCTGGAAGATGGTGAT-3’		
HTLV-1 Tax-F	5’-CACCTGTCCAGAGCATCAGA-3’	95 °C × 15 min; 45 cycles: 94°C × 30 s; 57 °C × 30s; 75 °C × 30 s; 75°C × 7 min	264
HTLV-1 Tax-R	5’-TCTGGAAAAGACAGGGTTGG-3’		

**Table 2:** Analysis of the association between HTLV-1 and HPV infections.

	HPV (+)	HPV (−)
**HTLV-1 (+)**	13	13
**HTLV-1 (−)**	108	89
***p*-value**	> 0.05	

## Supplementary Materials

The following are available online at www.mdpi.com/1999-4915/8/9/245/s1, Figure S1: PCR Analysis of HTLV-1 Tax in 220 LBC DNA’s, Figure S2: PCR Analysis of HTLV-1 Tax in 77 ICC DNA’s, Figure S3: HTLV-1 prevalence and cervical cytology in HIV+ve and −ve patients, Figure S4: Nested PCR analysis of the Tax 181 pb amplimer in the 26 HTLV-1 positive LBC’s, Table S1: Relationship between HTLV-1 and WHO HIV staging and HAART, Table S2: HTLV-1 status with respect to patient sociodemographics, Supplementary_Data_S2: NCBI BLAST of nested Tax amplimer sequences shown in supplementary Figure S4.
