# Supplementary material for: Analysis of the Prevalence of HTLV-1 Proviral DNA in Cervical Smears and Carcinomas from HIV Positive and Negative Kenyan Women
Source: Viruses. 2016 Sep 5;8(9):245. doi: 10.3390/v8090245 (PMC5035959; doi:10.3390/v8090245)
Supplement: Supplementary file 1 [file viruses-08-00245-s001.zip › viruses-115666-Supplementary_Data_S2.pdf]

NCBI BLAST of Nested Tax Amplimer Sequences Shown in Supplementary Figure 4

1. Human T-lymphotropic virus 1 isolate LDAA_04445_IC tax protein (tax) and rex protein (rex) genes, partial cds

Sequence ID: gb|KU680636.1|Length: 382Number of Matches: 1

Related Information
Range 1: 199 to 367

Score	Expect	Identities	Gaps	Strand
313 bits(169)	9e-82	169/169(100%)	0/169(0%)	Plus/Plus

```
Query  10   CCACCCAGAGAACCTCTAAGACCCTCAAGGTCCTTACCCCGCCAATCACTCATACAACCC  69
            ||||||||||||||||||||||||||||||||||||||||||||||||||||||||||||
Sbjct  199  CCACCCAGAGAACCTCTAAGACCCTCAAGGTCCTTACCCCGCCAATCACTCATACAACCC  258

Query  70   CCAACATTCCACCCTCCTTCCTCCAGGCCATGCGCAAATACTCCCCCTTCCGAAATGGAT  129
            ||||||||||||||||||||||||||||||||||||||||||||||||||||||||||||
Sbjct  259  CCAACATTCCACCCTCCTTCCTCCAGGCCATGCGCAAATACTCCCCCTTCCGAAATGGAT  318

Query  130  ACATGGAACCCACCCTTGGGCAGCACCTCCCAACCCTGTCTTTTCCAGA  178
            |||||||||||||||||||||||||||||||||||||||||||||||||
Sbjct  319  ACATGGAACCCACCCTTGGGCAGCACCTCCCAACCCTGTCTTTTCCAGA  367
```

2. Human T-lymphotropic virus 1 isolate LDAA_04445_IC tax protein (tax) and rex protein (rex) genes, partial cds

Sequence ID: gb|KU680636.1|Length: 382Number of Matches: 1

Related Information
Range 1: 202 to 367

Score	Expect	Identities	Gaps	Strand
302 bits(163)	2e-78	165/166(99%)	0/166(0%)	Plus/Plus

```
Query  13   CCCAGAGAACCTCTAAGACCCTCAAGGTCCTTACCCCGCCAATCACTCATACAACCCCAA  72
            |||||||||||||||||||||||||||||||||||||||||||||||||||||||||| |
Sbjct  202  CCCAGAGAACCTCTAAGACCCTCAAGGTCCTTACCCCGCCAATCACTCATACAACCCCCA  261

Query  73   ACATTCCACCCTCCTTCCTCCAGGCCATGCGCAAATACTCCCCCTTCCGAAATGGATACA  132
            ||||||||||||||||||||||||||||||||||||||||||||||||||||||||||||
Sbjct  262  ACATTCCACCCTCCTTCCTCCAGGCCATGCGCAAATACTCCCCCTTCCGAAATGGATACA  321

Query  133  TGGAACCCACCCTTGGGCAGCACCTCCCAACCCTGTCTTTTCCAGA  178
            ||||||||||||||||||||||||||||||||||||||||||||||
Sbjct  322  TGGAACCCACCCTTGGGCAGCACCTCCCAACCCTGTCTTTTCCAGA  367
```

3. Human T-lymphotropic virus 1 isolate LDAA_04445_IC tax protein (tax) and rex protein (rex) genes, partial cds

Sequence ID: gb|KU680636.1|Length: 382Number of Matches: 1

Related Information
Range 1: 203 to 367

Score	Expect	Identities	Gaps	Strand
257 bits(139)	4e-65	156/166(94%)	2/166(1%)	Plus/Plus

```
Query  13   CCAGAG-ACCTCTAANACCCTCAAGGNCCTTACCCCGCCAATCACTCATACCATNNCCAA  71
            |||||| |||||||| |||||||||| |||||||||||||||||||||||| |   ||||
Sbjct  203  CCAGAGAACCTCTAAGACCCTCAAGGTCCTTACCCCGCCAATCACTCATACAACCCCCAA  262

Query  72   AAGTTCCACCCTCCTTCCTCCAGGCCATGCGCAAATACTCTCCCTTCCGAAATGGATACA  131
             | ||||||||||||||||||||||||||||||||||||| |||||||||||||||||||
Sbjct  263  CA-TTCCACCCTCCTTCCTCCAGGCCATGCGCAAATACTCCCCCTTCCGAAATGGATACA  321

Query  132  TGGAACCCACCCTTGGGCAGCACCTCCCAACCCTGTCTTTTCCAGA  177
            ||||||||||||||||||||||||||||||||||||||||||||||
Sbjct  322  TGGAACCCACCCTTGGGCAGCACCTCCCAACCCTGTCTTTTCCAGA  367
```

4. Human T-lymphotropic virus 1 isolate LDAA_04733_IC tax protein (tax) and rex protein (rex) genes, partial cds

Sequence ID: gb|KU680637.1|Length: 382Number of Matches: 1

Related Information

Range 1: 189 to 367

Score	Expect	Identities	Gaps	Strand
190 bits(210)	7e-45	151/180(84%)	4/180(2%)	Plus/Plus

```
Query  2    CCCTCCTTCCCC--CCAGAGAACCTCTAANACCCNTACCGNCGTTGCCCCGCCAATCACT  59
            ||||||||||||  ||||||||||||||| |||| ||  | | || ||||||||||||||
Sbjct  189  CCCTCCTTCCCCACCCAGAGAACCTCTAAGACCCTTAAAGTCCTTACCCCGCCAATCACT  248

Query  60   NATACCAACCANGGAAGNGCCACACTCCTTCCTCCAGGCCATGNGCAAATACCCCCCCTT  119
             |||| ||||    |    |||| ||||||||||||||||||| |||||||| |||||||
Sbjct  249  CATAC-AACCCCCAACATTCCACCCTCCTTCCTCCAGGCCATGCGCAAATACTCCCCCTT  307

Query  120  CCCAAATGGATACATGGAA-CTACCCTTGGGCAGCACCTTTCAACCCTGTCTTTCCCCGA  178
            || |||||||||||||||| | |||||||||||||||||  ||||||||||||| || ||
Sbjct  308  CCGAAATGGATACATGGAACCCACCCTTGGGCAGCACCTCCCAACCCTGTCTTTTCCAGA  367
```

5. Human T-lymphotropic virus 1 isolate LDAA_03998_IC tax protein (tax) and rex protein (rex) genes, partial cds

Sequence ID: gb|KU680633.1|Length: 382Number of Matches: 1

Related Information

Range 1: 199 to 367

Score	Expect	Identities	Gaps	Strand
275 bits(304)	2e-70	163/170(96%)	1/170(0%)	Plus/Plus

```
Query  8    CCACCCAGAGAACCTCTAAGACCCTTAAGGTCCTTACCCCGCCAATCACTCATACCANNC  67
            ||||||||||||||||||||||||||||||||||||||||||||||||||||||| |  |
Sbjct  199  CCACCCAGAGAACCTCTAAGACCCTTAAGGTCCTTACCCCGCCAATCACTCATACAACCC  258

Query  68   naaaaaaTTCCACCCTCCTTCCTCCAGGCCATGCGCAAATACTCCCCCTTCCGAAATGGA  127
              || | |||||||||||||||||||||||||||||||||||||||||||||||||||||
Sbjct  259  CCAACA-TTCCACCCTCCTTCCTCCAGGCCATGCGCAAATACTCCCCCTTCCGAAATGGA  317

Query  128  TACATGGAACCCACCCTTGGGCAGCACCTCCCAACCCTGTCTTTTCCAGA  177
            ||||||||||||||||||||||||||||||||||||||||||||||||||
Sbjct  318  TACATGGAACCCACCCTTGGGCAGCACCTCCCAACCCTGTCTTTTCCAGA  367
```

6. Human T-lymphotropic virus 1 isolate BRSP217-12 tax protein (tax) gene, partial cds

Sequence ID: gb|KT351510.1|Length: 996Number of Matches: 1

Related Information

Range 1: 173 to 345

Score	Expect	Identities	Gaps	Strand
152 bits(168)	2e-33	141/175(81%)	8/175(4%)	Plus/Plus

```
Query  6    CCACCAAAAGANCC-CTAAGACCCTCCAGGTCCTTATAACGC-AATTACTCATANANANC  63
            ||||| | ||| || ||||||||||| |||||||||   ||| ||| ||||||| |   |
Sbjct  173  CCACCCAGAGAACCTCTAAGACCCTCAAGGTCCTTACCCCGCCAATCACTCATACAACCC  232

Query  64   NCGA-ANNCNACCCTTTTT--TCAAGGC-ATGCGCAAANATTCCATNAATTCCGAAATGG  119
             | | |  | |||||  ||  || |||| ||||||||| | |||     |||||||||||
Sbjct  233  CCAACATTCCACCCTCCTTCCTCCAGGCCATGCGCAAATACTCCCCC--TTCCGAAATGG  290

Query  120  ATACATGGAACCCACCCTTGGGCAGCACCTCCCAACCCTGTCTTTCCCAGAACCC  174
            ||||||||||||||||||||||||||||||||||||||||||||||||||| |||
Sbjct  291  ATACATGGAACCCACCCTTGGGCAGCACCTCCCAACCCTGTCTTTCCCAGACCCC  345
```

7. Human T-lymphotropic virus 1 isolate LDAA_04272_IC tax protein (tax) and rex protein (rex) genes, partial cds

Sequence ID: gb|KU680634.1|Length: 382Number of Matches: 1

Related Information

Range 1: 200 to 367

Score	Expect	Identities	Gaps	Strand
304 bits(336)	4e-79	168/168(100%)	0/168(0%)	Plus/Plus

```
Query  1    CACCCAGAGAACCTCTAAGACCCTCAAGGTCCTTACCCCGCCAATCACTCATACAACCCC  60
            ||||||||||||||||||||||||||||||||||||||||||||||||||||||||||||
Sbjct  200  CACCCAGAGAACCTCTAAGACCCTCAAGGTCCTTACCCCGCCAATCACTCATACAACCCC  259

Query  61   CAACATTCCACCCTCCTTCCTCCAGGCCATGCGCAAATACTCCCCCTTCCGAAATGGATA  120
            ||||||||||||||||||||||||||||||||||||||||||||||||||||||||||||
Sbjct  260  CAACATTCCACCCTCCTTCCTCCAGGCCATGCGCAAATACTCCCCCTTCCGAAATGGATA  319

Query  121  CATGGAACCCACCCTTGGGCAGCACCTCCCAACCCTGTCTTTTCCAGA  168
            ||||||||||||||||||||||||||||||||||||||||||||||||
Sbjct  320  CATGGAACCCACCCTTGGGCAGCACCTCCCAACCCTGTCTTTTCCAGA  367
```

8. Human T-lymphotropic virus 1 isolate LDAA_02481_IC tax protein (tax) and rex protein (rex) genes, partial cds

Sequence ID: gb|KU680629.1|Length: 382Number of Matches: 1

Related Information

Range 1: 282 to 371

Score	Expect	Identities	Gaps	Strand
125 bits(138)	3e-25	84/92(91%)	3/92(3%)	Plus/Plus

```
Query  85   CAGGCC-TGCGCAAAccccccccnGGTTTCGAAATGGATACATGGAACCCACCCTTGGGC  143
            |||||| ||||||||| | ||||   || |||||||||||||||||||||||||||||||
Sbjct  282  CAGGCCATGCGCAAACACTCCCCC--TTCCGAAATGGATACATGGAACCCACCCTTGGGC  339

Query  144  AGCACCTCCCAACCCTGTCTTTTCCAGAACCC  175
            |||||||||||||||||||||||||||| |||
Sbjct  340  AGCACCTCCCAACCCTGTCTTTTCCAGACCCC  371
```

9. Human T-lymphotropic virus 1 isolate LDAA_04445_IC tax protein (tax) and rex protein (rex) genes, partial cds

Sequence ID: gb|KU680636.1|Length: 382Number of Matches: 1

Related Information

Range 1: 188 to 367

Score	Expect	Identities	Gaps	Strand
315 bits(348)	3e-82	179/181(99%)	1/181(0%)	Plus/Plus

```
Query  1    CCCCTCCTTccccnccccAGAGAACCTCTAAGACCCTCAAGGTCCTTACCCCGCCAATCA  60
            ||||||||||||| ||| ||||||||||||||||||||||||||||||||||||||||||
Sbjct  188  CCCCTCCTTCCCCACCC-AGAGAACCTCTAAGACCCTCAAGGTCCTTACCCCGCCAATCA  246

Query  61   CTCATACAACCCCCAACATTCCACCCTCCTTCCTCCAGGCCATGCGCAAATACTCCCCCT  120
            ||||||||||||||||||||||||||||||||||||||||||||||||||||||||||||
Sbjct  247  CTCATACAACCCCCAACATTCCACCCTCCTTCCTCCAGGCCATGCGCAAATACTCCCCCT  306

Query  121  TCCGAAATGGATACATGGAACCCACCCTTGGGCAGCACCTCCCAACCCTGTCTTTTCCAG  180
            ||||||||||||||||||||||||||||||||||||||||||||||||||||||||||||
Sbjct  307  TCCGAAATGGATACATGGAACCCACCCTTGGGCAGCACCTCCCAACCCTGTCTTTTCCAG  366

Query  181  A  181
            |
Sbjct  367  A  367
```

10. Human T-lymphotropic virus 1 strain BRRJ3476 tax protein (tax) gene, partial cds

Sequence ID: gb|DQ323834.1|Length: 1000Number of Matches: 1

Related Information

Range 1: 208 to 374

Score	Expect	Identities	Gaps	Strand
147 bits(162)	8e-32	135/169(80%)	10/169(5%)	Plus/Plus

```
Query  16   AGAGAACCTCTCAGACNNTNA-GGNTCTTACCCCGTCCAATCACTCATAANNCCCCGCAN  74
            ||||||||||| ||||  | | ||  ||||||||| |||||||||||||   ||||| |
Sbjct  208  AGAGAACCTCTAAGACCCTCAAGGGCCTTACCCCG-CCAATCACTCATACAACCCCGAAC  266

Query  75   NTAACTC------TTCTCCAGGC-ATGCGGAAANAATCTCAAATTCCGAAATGGATACAT  127
             |  | |      | |||||||| ||||| ||| | || |   |||||||||||||||||
Sbjct  267  ATTCCACCCTCCTTCCTCCAGGCCATGCGCAAATACTCCCCC-TTCCGAAATGGATACAT  325

Query  128  GGAACCCACCCTTGGGCAGTACCTCCCAACCCTGTCTTTTCCAGAACCC  176
            ||||||||||||||||||| ||||||||||||||||||||||||| |||
Sbjct  326  GGAACCCACCCTTGGGCAGCACCTCCCAACCCTGTCTTTTCCAGACCCC  374
```

11. Human T-lymphotropic virus 1 isolate LDAA_04445_IC tax protein (tax) and rex protein (rex) genes, partial cds

Sequence ID: gb|KU680636.1|Length: 382Number of Matches: 1

Related Information

Range 1: 192 to 367

Score	Expect	Identities	Gaps	Strand
289 bits(320)	1e-74	170/176(97%)	1/176(0%)	Plus/Plus

```
Query  1    TCCTTCCC-ACCCAGAGAACCTCTAAGACCCTCAAGGTCCTTACCCCGCCAATCACTCAT  59
            |||||||| |||||||||||||||||||||||||||||||||||||||||||||||||||
Sbjct  192  TCCTTCCCCACCCAGAGAACCTCTAAGACCCTCAAGGTCCTTACCCCGCCAATCACTCAT  251

Query  60   ACANNAACCAAAATTCCACCCTCCTTCCTCCAGGCCATGCGCAAATACTCCCCCTTCCGA  119
            |||    |||| ||||||||||||||||||||||||||||||||||||||||||||||||
Sbjct  252  ACAACCCCCAACATTCCACCCTCCTTCCTCCAGGCCATGCGCAAATACTCCCCCTTCCGA  311

Query  120  AATGGATACATGGAACCCACCCTTGGGCAGCACCTCCCAACCCTGTCTTTTCCAGA  175
            ||||||||||||||||||||||||||||||||||||||||||||||||||||||||
Sbjct  312  AATGGATACATGGAACCCACCCTTGGGCAGCACCTCCCAACCCTGTCTTTTCCAGA  367
```

12. Human T-lymphotropic virus 1 isolate LDAA_04733_IC tax protein (tax) and rex protein (rex) genes, partial cds

Sequence ID: gb|KU680637.1|Length: 382Number of Matches: 1

Related Information

Range 1: 303 to 367

Score	Expect	Identities	Gaps	Strand
66.2 bits(72)	5e-08	53/65(82%)	0/65(0%)	Plus/Plus

```
Query  5    CCATTTCGAAATGGATACATGGAAACNTCNNTTGGGCAGGAACTCTTAACCCTGGCTTTT  64
            || || |||||||||||||||||| |  |  |||||||| | |||  ||||||| |||||
Sbjct  303  CCCTTCCGAAATGGATACATGGAACCCACCCTTGGGCAGCACCTCCCAACCCTGTCTTTT  362

Query  65   CCAGA  69
            |||||
Sbjct  363  CCAGA  367
```

13. Human T-lymphotropic virus 1 isolate LDAA_04445_IC tax protein (tax) and rex protein (rex) genes, partial cds
Sequence ID: gb|KU680636.1|Length: 382Number of Matches: 1
Related Information
Range 1: 192 to 370

Score	Expect	Identities	Gaps	Strand
233 bits(258)	7e-58	162/182(89%)	3/182(1%)	Plus/Plus

```
Query  1    TCCTTCTTCCACCTAGAGAACCTCTAAGAACCTTCAAGGTCCTTACCCCGCCAATCACTC  60
            ||||||  ||||| ||||||||||||||| ||| ||||||||||||||||||||||||||
Sbjct  192  TCCTTCC-CCACCCAGAGAACCTCTAAGACCCT-CAAGGTCCTTACCCCGCCAATCACTC  249

Query  61   ATACANNNAAAAAANTTNCACCCTCCTTCCTCCAGGCCATGCGCAAATACCTNTCCCTTC  120
            |||||      ||  || |||||||||||||||||||||||||||||||| |  ||||||
Sbjct  250  ATACAACCCCCAACATTCCACCCTCCTTCCTCCAGGCCATGCGCAAATAC-TCCCCCTTC  308

Query  121  CGAAATGGATACATGGAACCTACCCTTGGGCAGCACCTCTCAACCCTGTCTTTTCCAGAA  180
            |||||||||||||||||||| |||||||||||||||||| ||||||||||||||||||| 
Sbjct  309  CGAAATGGATACATGGAACCCACCCTTGGGCAGCACCTCCCAACCCTGTCTTTTCCAGAC  368

Query  181  CC  182
            ||
Sbjct  369  CC  370
```

14. Human T-lymphotropic virus 1 isolate LDAA_04733_IC tax protein (tax) and rex protein (rex) genes, partial cds
Sequence ID: gb|KU680637.1|Length: 382Number of Matches: 1
Related Information
Range 1: 265 to 337

Score	Expect	Identities	Gaps	Strand
91.5 bits(100)	4e-15	62/73(85%)	0/73(0%)	Plus/Plus

```
Query  35   TTNCACCNTNCTTCCTCCAGGCCATGCGCAANNNNNNCCCCTTCCGAAATGGATACATGG  94
            || |||| | |||||||||||||||||||||      |||||||||||||||||||||||
Sbjct  265  TTCCACCCTCCTTCCTCCAGGCCATGCGCAAATACTCCCCCTTCCGAAATGGATACATGG  324

Query  95   AACCCANCTTTGG  107
            |||||| | ||||
Sbjct  325  AACCCACCCTTGG  337
```

15. Human T-lymphotropic virus 1 isolate LDAA_04445_IC tax protein (tax) and rex protein (rex) genes, partial cds
Sequence ID: gb|KU680636.1|Length: 382Number of Matches: 1
Related Information
Range 1: 202 to 365

Score	Expect	Identities	Gaps	Strand
297 bits(328)	6e-77	164/164(100%)	0/164(0%)	Plus/Plus

```
Query  1    CCCAGAGAACCTCTAAGACCCTCAAGGTCCTTACCCCGCCAATCACTCATACAACCCCCA  60
            ||||||||||||||||||||||||||||||||||||||||||||||||||||||||||||
Sbjct  202  CCCAGAGAACCTCTAAGACCCTCAAGGTCCTTACCCCGCCAATCACTCATACAACCCCCA  261

Query  61   ACATTCCACCCTCCTTCCTCCAGGCCATGCGCAAATACTCCCCCTTCCGAAATGGATACA  120
            ||||||||||||||||||||||||||||||||||||||||||||||||||||||||||||
Sbjct  262  ACATTCCACCCTCCTTCCTCCAGGCCATGCGCAAATACTCCCCCTTCCGAAATGGATACA  321

Query  121  TGGAACCCACCCTTGGGCAGCACCTCCCAACCCTGTCTTTTCCA  164
            ||||||||||||||||||||||||||||||||||||||||||||
Sbjct  322  TGGAACCCACCCTTGGGCAGCACCTCCCAACCCTGTCTTTTCCA  365
```

16. Human T-lymphotropic virus 1 isolate LDAA_04445_IC tax protein (tax) and rex protein (rex) genes, partial cds

Sequence ID: gb|KU680636.1|Length: 382Number of Matches: 1

Related Information
Range 1: 198 to 367

Score	Expect	Identities	Gaps	Strand
295 bits(326)	2e-76	168/170(99%)	1/170(0%)	Plus/Plus

```
Query  7    CCCACCCAGAGANC-TCTAAGACCCTCAAGGTCCTTACCCCGCCAATCACTCATACAACC  65
            |||||||||||| | ||||||||||||||||||||||||||||||||||||||||||||||
Sbjct  198  CCCACCCAGAGAACCTCTAAGACCCTCAAGGTCCTTACCCCGCCAATCACTCATACAACC  257

Query  66   CCCAACATTCCACCCTCCTTCCTCCAGGCCATGCGCAAATACTCCCCCTTCCGAAATGGA  125
            ||||||||||||||||||||||||||||||||||||||||||||||||||||||||||||
Sbjct  258  CCCAACATTCCACCCTCCTTCCTCCAGGCCATGCGCAAATACTCCCCCTTCCGAAATGGA  317

Query  126  TACATGGAACCCACCCTTGGGCAGCACCTCCCAACCCTGTCTTTTCCAGA  175
            ||||||||||||||||||||||||||||||||||||||||||||||||||
Sbjct  318  TACATGGAACCCACCCTTGGGCAGCACCTCCCAACCCTGTCTTTTCCAGA  367
```

17. Human T-lymphotropic virus 1 isolate LDAA_04445_IC tax protein (tax) and rex protein (rex) genes, partial cds

Sequence ID: gb|KU680636.1|Length: 382Number of Matches: 1

Related Information
Range 1: 198 to 367

Score	Expect	Identities	Gaps	Strand
307 bits(340)	4e-80	170/170(100%)	0/170(0%)	Plus/Plus

```
Query  5    CCCACCCAGAGAACCTCTAAGACCCTCAAGGTCCTTACCCCGCCAATCACTCATACAACC  64
            ||||||||||||||||||||||||||||||||||||||||||||||||||||||||||||
Sbjct  198  CCCACCCAGAGAACCTCTAAGACCCTCAAGGTCCTTACCCCGCCAATCACTCATACAACC  257

Query  65   CCCAACATTCCACCCTCCTTCCTCCAGGCCATGCGCAAATACTCCCCCTTCCGAAATGGA  124
            ||||||||||||||||||||||||||||||||||||||||||||||||||||||||||||
Sbjct  258  CCCAACATTCCACCCTCCTTCCTCCAGGCCATGCGCAAATACTCCCCCTTCCGAAATGGA  317

Query  125  TACATGGAACCCACCCTTGGGCAGCACCTCCCAACCCTGTCTTTTCCAGA  174
            ||||||||||||||||||||||||||||||||||||||||||||||||||
Sbjct  318  TACATGGAACCCACCCTTGGGCAGCACCTCCCAACCCTGTCTTTTCCAGA  367
```

18. Human T-lymphotropic virus 1 isolate LDAA_04272_IC tax protein (tax) and rex protein (rex) genes, partial cds

Sequence ID: gb|KU680634.1|Length: 382Number of Matches: 1

Related Information
Range 1: 200 to 367

Score	Expect	Identities	Gaps	Strand
304 bits(336)	5e-79	168/168(100%)	0/168(0%)	Plus/Plus

```
Query  11   CACCCAGAGAACCTCTAAGACCCTCAAGGTCCTTACCCCGCCAATCACTCATACAACCCC  70
            ||||||||||||||||||||||||||||||||||||||||||||||||||||||||||||
Sbjct  200  CACCCAGAGAACCTCTAAGACCCTCAAGGTCCTTACCCCGCCAATCACTCATACAACCCC  259

Query  71   CAACATTCCACCCTCCTTCCTCCAGGCCATGCGCAAATACTCCCCCTTCCGAAATGGATA  130
            ||||||||||||||||||||||||||||||||||||||||||||||||||||||||||||
Sbjct  260  CAACATTCCACCCTCCTTCCTCCAGGCCATGCGCAAATACTCCCCCTTCCGAAATGGATA  319

Query  131  CATGGAACCCACCCTTGGGCAGCACCTCCCAACCCTGTCTTTTCCAGA  178
            ||||||||||||||||||||||||||||||||||||||||||||||||
Sbjct  320  CATGGAACCCACCCTTGGGCAGCACCTCCCAACCCTGTCTTTTCCAGA  367
```

19. Human T-lymphotropic virus 1 isolate LDAA_03584_IC tax protein (tax) and rex protein (rex) genes, partial cds

Sequence ID: gb|KU680631.1|Length: 382Number of Matches: 1
Related Information
Range 1: 189 to 367

Score	Expect	Identities	Gaps	Strand
320 bits(173)	6e-84	177/179(99%)	1/179(0%)	Plus/Plus

```
Query  2    CCCTCCTT-CNCACCCAGAGAACCTCTAAGACCCTCAAGGTCCTTACCCCGCCAATCACT  60
            |||||||| | |||||||||||||||||||||||||||||||||||||||||||||||||
Sbjct  189  CCCTCCTTCCCCACCCAGAGAACCTCTAAGACCCTCAAGGTCCTTACCCCGCCAATCACT  248

Query  61   CATACAACCCCCAACATTCCACCCTCCTTCCTCCAGGCCATGCGCAAATACTCCCCCTTC  120
            ||||||||||||||||||||||||||||||||||||||||||||||||||||||||||||
Sbjct  249  CATACAACCCCCAACATTCCACCCTCCTTCCTCCAGGCCATGCGCAAATACTCCCCCTTC  308

Query  121  CGAAATGGATACATGGAACCCACCCTTGGGCAGCACCTCCCAACCCTGTCTTTTCCAGA  179
            |||||||||||||||||||||||||||||||||||||||||||||||||||||||||||
Sbjct  309  CGAAATGGATACATGGAACCCACCCTTGGGCAGCACCTCCCAACCCTGTCTTTTCCAGA  367
```

20. Human T-lymphotropic virus 1 isolate LDAA_04445_IC tax protein (tax) and rex protein (rex) genes, partial cds

Sequence ID: gb|KU680636.1|Length: 382Number of Matches: 1
Related Information
Range 1: 190 to 367

Score	Expect	Identities	Gaps	Strand
316 bits(171)	7e-83	176/178(99%)	2/178(1%)	Plus/Plus

```
Query  3    CCT-CTT-CCCACCCAGAGAACCTCTAAGACCCTCAAGGTCCTTACCCCGCCAATCACTC  60
            ||| ||| ||||||||||||||||||||||||||||||||||||||||||||||||||||
Sbjct  190  CCTCCTTCCCCACCCAGAGAACCTCTAAGACCCTCAAGGTCCTTACCCCGCCAATCACTC  249

Query  61   ATACAACCCCCAACATTCCACCCTCCTTCCTCCAGGCCATGCGCAAATACTCCCCCTTCC  120
            ||||||||||||||||||||||||||||||||||||||||||||||||||||||||||||
Sbjct  250  ATACAACCCCCAACATTCCACCCTCCTTCCTCCAGGCCATGCGCAAATACTCCCCCTTCC  309

Query  121  GAAATGGATACATGGAACCCACCCTTGGGCAGCACCTCCCAACCCTGTCTTTTCCAGA  178
            ||||||||||||||||||||||||||||||||||||||||||||||||||||||||||
Sbjct  310  GAAATGGATACATGGAACCCACCCTTGGGCAGCACCTCCCAACCCTGTCTTTTCCAGA  367
```

21. Human T-lymphotropic virus 1 isolate LDAA_04445_IC tax protein (tax) and rex protein (rex) genes, partial cds

Sequence ID: gb|KU680636.1|Length: 382Number of Matches: 1
Related Information
Range 1: 185 to 367

Score	Expect	Identities	Gaps	Strand
311 bits(168)	3e-81	178/183(97%)	3/183(1%)	Plus/Plus

```
Query  1    ACT-CCNTCCTT-CCCACCCAGAG-ANCTCTAAGACCCTCAAGGTCCTTACCCCGCCAAT  57
            ||| || ||||| ||||||||||| | |||||||||||||||||||||||||||||||||
Sbjct  185  ACTCCCCTCCTTCCCCACCCAGAGAACCTCTAAGACCCTCAAGGTCCTTACCCCGCCAAT  244

Query  58   CACTCATACAACCCCCAACATTCCACCCTCCTTCCTCCAGGCCATGCGCAAATACTCCCC  117
            ||||||||||||||||||||||||||||||||||||||||||||||||||||||||||||
Sbjct  245  CACTCATACAACCCCCAACATTCCACCCTCCTTCCTCCAGGCCATGCGCAAATACTCCCC  304

Query  118  CTTCCGAAATGGATACATGGAACCCACCCTTGGGCAGCACCTCCCAACCCTGTCTTTTCC  177
            ||||||||||||||||||||||||||||||||||||||||||||||||||||||||||||
Sbjct  305  CTTCCGAAATGGATACATGGAACCCACCCTTGGGCAGCACCTCCCAACCCTGTCTTTTCC  364

Query  178  AGA  180
            |||
Sbjct  365  AGA  367
```

22. Human T-lymphotropic virus 1 isolate LDAA_04445_IC tax protein (tax) and rex protein (rex) genes, partial cds

Sequence ID: gb|KU680636.1|Length: 382Number of Matches: 1
Related Information
Range 1: 187 to 367

Score	Expect	Identities	Gaps	Strand
322 bits(174)	2e-84	179/181(99%)	2/181(1%)	Plus/Plus

```
Query  1    TCCCCT-CTT-CCCACCCAGAGAACCTCTAAGACCCTCAAGGTCCTTACCCCGCCAATCA  58
            |||||| ||| |||||||||||||||||||||||||||||||||||||||||||||||||
Sbjct  187  TCCCCTCCTTCCCCACCCAGAGAACCTCTAAGACCCTCAAGGTCCTTACCCCGCCAATCA  246

Query  59   CTCATACAACCCCCAACATTCCACCCTCCTTCCTCCAGGCCATGCGCAAATACTCCCCCT  118
            ||||||||||||||||||||||||||||||||||||||||||||||||||||||||||||
Sbjct  247  CTCATACAACCCCCAACATTCCACCCTCCTTCCTCCAGGCCATGCGCAAATACTCCCCCT  306

Query  119  TCCGAAATGGATACATGGAACCCACCCTTGGGCAGCACCTCCCAACCCTGTCTTTTCCAG  178
            ||||||||||||||||||||||||||||||||||||||||||||||||||||||||||||
Sbjct  307  TCCGAAATGGATACATGGAACCCACCCTTGGGCAGCACCTCCCAACCCTGTCTTTTCCAG  366

Query  179  A  179
            |
Sbjct  367  A  367
```

23. Human T-lymphotropic virus 1 isolate LDAA_03584_IC tax protein (tax) and rex protein (rex) genes, partial cds
Sequence ID: gb|KU680631.1|Length: 382Number of Matches: 1
Related Information
Range 1: 190 to 367

Score	Expect	Identities	Gaps	Strand
316 bits(171)	7e-83	176/178(99%)	2/178(1%)	Plus/Plus

```
Query  2    CCT-CTT-CCCACCCAGAGAACCTCTAAGACCCTCAAGGTCCTTACCCCGCCAATCACTC  59
            ||| ||| ||||||||||||||||||||||||||||||||||||||||||||||||||||
Sbjct  190  CCTCCTTCCCCACCCAGAGAACCTCTAAGACCCTCAAGGTCCTTACCCCGCCAATCACTC  249

Query  60   ATACAACCCCCAACATTCCACCCTCCTTCCTCCAGGCCATGCGCAAATACTCCCCCTTCC  119
            ||||||||||||||||||||||||||||||||||||||||||||||||||||||||||||
Sbjct  250  ATACAACCCCCAACATTCCACCCTCCTTCCTCCAGGCCATGCGCAAATACTCCCCCTTCC  309

Query  120  GAAATGGATACATGGAACCCACCCTTGGGCAGCACCTCCCAACCCTGTCTTTTCCAGA  177
            ||||||||||||||||||||||||||||||||||||||||||||||||||||||||||
Sbjct  310  GAAATGGATACATGGAACCCACCCTTGGGCAGCACCTCCCAACCCTGTCTTTTCCAGA  367
```

24. Human T-lymphotropic virus 1 isolate LDAA_04272_IC tax protein (tax) and rex protein (rex) genes, partial cds
Sequence ID: gb|KU680634.1|Length: 382Number of Matches: 1
Related Information
Range 1: 192 to 367

Score	Expect	Identities	Gaps	Strand
318 bits(172)	2e-83	175/176(99%)	1/176(0%)	Plus/Plus

```
Query  1    TCCTT-CCCACCCAGAGAACCTCTAAGACCCTCAAGGTCCTTACCCCGCCAATCACTCAT  59
            ||||| ||||||||||||||||||||||||||||||||||||||||||||||||||||||
Sbjct  192  TCCTTCCCCACCCAGAGAACCTCTAAGACCCTCAAGGTCCTTACCCCGCCAATCACTCAT  251

Query  60   ACAACCCCCAACATTCCACCCTCCTTCCTCCAGGCCATGCGCAAATACTCCCCCTTCCGA  119
            ||||||||||||||||||||||||||||||||||||||||||||||||||||||||||||
Sbjct  252  ACAACCCCCAACATTCCACCCTCCTTCCTCCAGGCCATGCGCAAATACTCCCCCTTCCGA  311

Query  120  AATGGATACATGGAACCCACCCTTGGGCAGCACCTCCCAACCCTGTCTTTTCCAGA  175
            ||||||||||||||||||||||||||||||||||||||||||||||||||||||||
Sbjct  312  AATGGATACATGGAACCCACCCTTGGGCAGCACCTCCCAACCCTGTCTTTTCCAGA  367
```

25. Human T-lymphotropic virus 1 isolate LDAA_04445_IC tax protein (tax) and rex protein (rex) genes, partial cds

Sequence ID: gb|KU680636.1|Length: 382Number of Matches: 1
Related Information
Range 1: 200 to 367

Score	Expect	Identities	Gaps	Strand
311 bits(168)	3e-81	168/168(100%)	0/168(0%)	Plus/Plus

```
Query  1    CACCCAGAGAACCTCTAAGACCCTCAAGGTCCTTACCCCGCCAATCACTCATACAACCCC  60
            ||||||||||||||||||||||||||||||||||||||||||||||||||||||||||||
Sbjct  200  CACCCAGAGAACCTCTAAGACCCTCAAGGTCCTTACCCCGCCAATCACTCATACAACCCC  259

Query  61   CAACATTCCACCCTCCTTCCTCCAGGCCATGCGCAAATACTCCCCCTTCCGAAATGGATA  120
            ||||||||||||||||||||||||||||||||||||||||||||||||||||||||||||
Sbjct  260  CAACATTCCACCCTCCTTCCTCCAGGCCATGCGCAAATACTCCCCCTTCCGAAATGGATA  319

Query  121  CATGGAACCCACCCTTGGGCAGCACCTCCCAACCCTGTCTTTTCCAGA  168
            ||||||||||||||||||||||||||||||||||||||||||||||||
Sbjct  320  CATGGAACCCACCCTTGGGCAGCACCTCCCAACCCTGTCTTTTCCAGA  367
```

26. Human T-lymphotropic virus 1 isolate LDAA_04445_IC tax protein (tax) and rex protein (rex) genes, partial cds
Sequence ID: gb|KU680636.1|Length: 382Number of Matches: 1
Related Information
Range 1: 198 to 367

Score	Expect	Identities	Gaps	Strand
315 bits(170)	2e-82	170/170(100%)	0/170(0%)	Plus/Plus

```
Query  1    CCCACCCAGAGAACCTCTAAGACCCTCAAGGTCCTTACCCCGCCAATCACTCATACAACC  60
            ||||||||||||||||||||||||||||||||||||||||||||||||||||||||||||
Sbjct  198  CCCACCCAGAGAACCTCTAAGACCCTCAAGGTCCTTACCCCGCCAATCACTCATACAACC  257

Query  61   CCCAACATTCCACCCTCCTTCCTCCAGGCCATGCGCAAATACTCCCCCTTCCGAAATGGA  120
            ||||||||||||||||||||||||||||||||||||||||||||||||||||||||||||
Sbjct  258  CCCAACATTCCACCCTCCTTCCTCCAGGCCATGCGCAAATACTCCCCCTTCCGAAATGGA  317

Query  121  TACATGGAACCCACCCTTGGGCAGCACCTCCCAACCCTGTCTTTTCCAGA  170
            ||||||||||||||||||||||||||||||||||||||||||||||||||
Sbjct  318  TACATGGAACCCACCCTTGGGCAGCACCTCCCAACCCTGTCTTTTCCAGA  367
```

These data show homology with the following HTLV-1 isolates:-
14 x LDAA_04445_IC; 3 x LDAA_04733_IC; 1 x LDAA_03998; 1 X BRSP217-12; 3 x LDAA_04272_IC; 1 x BRRJ3476; 1 x LDAA_02481_IC; 2 x LDAA_03584_IC
