# Supplementary material for: Analysis of the Prevalence of HTLV-1 Proviral DNA in Cervical Smears and Carcinomas from HIV Positive and Negative Kenyan Women
Source: Viruses. 2016 Sep 5;8(9):245. doi: 10.3390/v8090245 (PMC5035959; doi:10.3390/v8090245)
Supplement: Supplementary file 1 [file viruses-08-00245-s001.zip › viruses-115666-Supplementary_Data_S1.pdf]

Supplementary Materials: Analysis of the Prevalence of HTLV-1 Proviral DNA in Cervical Smears and Carcinomas from HIV Positive and Negative Kenyan Women

Xiaotong He, Innocent O. Maranga, Anthony W. Oliver, Peter Gichangi, Hampson L. and Ian N. Hampson

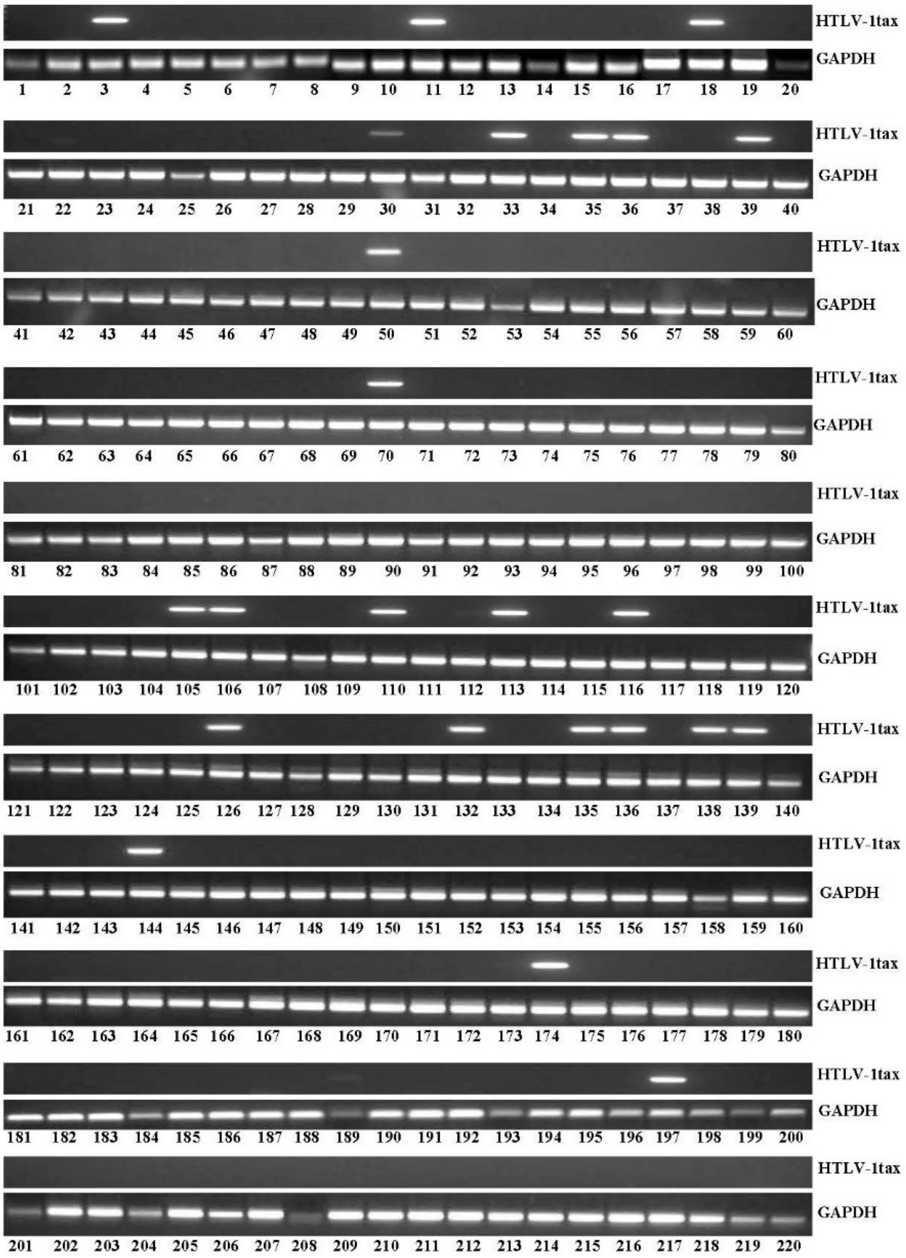

Figure S1. PCR Analysis of HTLV-1 Tax in 220 LBC DNA's.

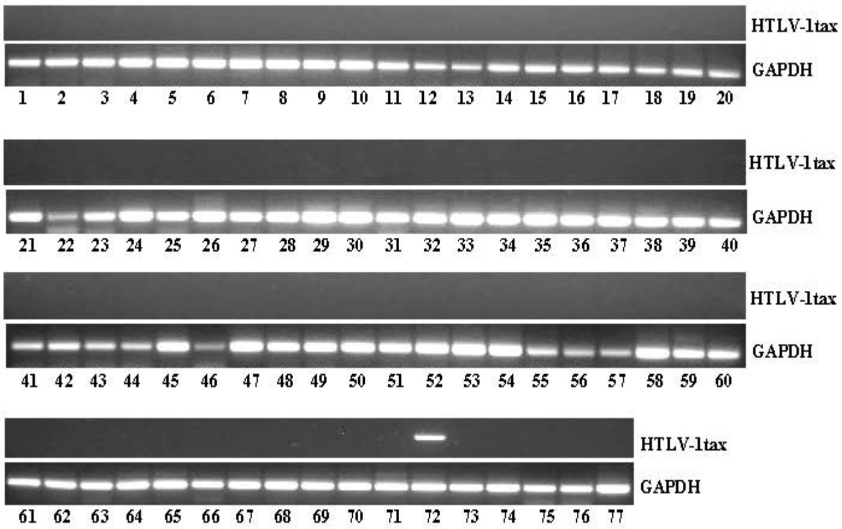

Figure S2. PCR Analysis of HTLV-1 Tax in 77 ICC DNA's.

Table S1. Relationship between HTLV-1 and WHO HIV staging and HAART.

	**WHO HIV Stage**				**HAART**		
	**I**	**II**	**III**	**IV**	**Yes**	**No**	**Total**
**HTLV1+ve**	6%/27.3%	1%/4.5%	6%/27.3%	9%/40.9%	12%/54.5%	10%/45.5%	22%/19.5%
**HTLV1−ve**	18%/19.8%	15%/16%	32%/35.2%	26%/28.5%	53%/58.2%	38%/41.8%	91%/80.5%
	24%/21.2%	16%/14.2%	38%/33.6%	35%/31.0%	65%/57.5%	48%/42.5%	113%/100%
***p*-value**	0.32				0.94		

Although there were more HTLV-1 infections in late HIV stages (III/IV) than early (I/II), this did not attain statistical significance ($p = 0.32$). Likewise, there was no signicant association between HTLV-1 infection and the use of HAART among HIV+ve women ($p = 0.94$).

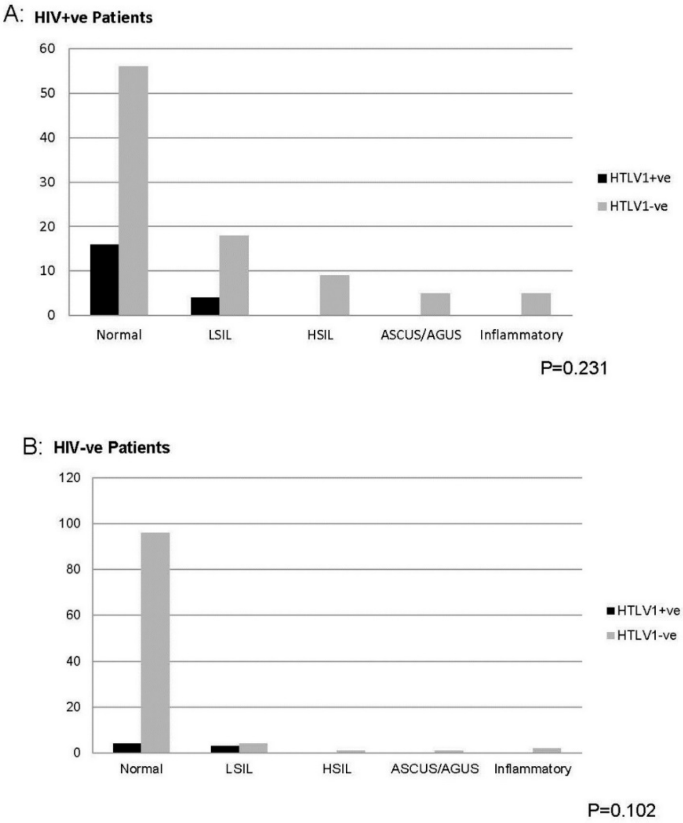

Figure S3. HTLV-1 prevalence and cervical cytology in HIV+ve and −ve patients.

Table S2. HTLV-1 status with respect to patient sociodemographics.

		HTLV-1+ve	HTLV-1–ve	*p*-Value
**Religion**	Catholic	10%/30.5%	53%/26.8%	0.39
	Protestant	16%/61.5%	131%/66.2%	
	Muslim	0%/0.0%	6%/3.0%	
	Other	0%/0.0%	8%/4.0%	
**Educational level**	None	0%/0.0%	2%/1.1%	0.87
	Primary	5%/19.2%	49%/24.7%	
	Secondary	9%/34.6%	60%/30.3%	
	College/Tertiary	12%/46.2%	87%/47.9%	
**Occupation**	Self-employed	5%/19.2%	25%/12.6%	0.64
	Formal employment	9%/34.6%	71%/35.9%	
	Business	5%/19.2%	61%/30.8%	
	Student	9%/34.6%	60%/30.3%	
	Unemployed	7%/27.0%	36%/18.2%	
	Other	0%/0.0%	3%/1.5%	
**Socio-econoic status**	Low	18%/69.2%	124%/62.6%	0.52
	Middle	5%/19.2%	58%/29.3%	
	High	3%/11.6%	16%/8.1%	
**Usual Residence**	Rural	3%/11,5%	37%/18.7%	0.62
	Urban	23%/88.5%	160%/80.8%	
	Foreigner	0%/0.0%	1%/0.5%	
**Contraceptive use**	Yes	14%/53.8%	109%/55.6%	1.00
	None	12%/46.2%	87%/44.4%	
**IV drug use**	Yes	0%/0.0%	3%/1.5%	1.00
	None	26%/100%	195%/98.5%	
**Blood transfusion**	Yes	1%/3.8%	13%/6.6%	0.91
	None	25%/96.2%	184%/93.4%	
**Genital herpes**	Yes	3%/11.5%	13%/6.6%	0.61
	None	23%/88.5%	184%/93.4%	

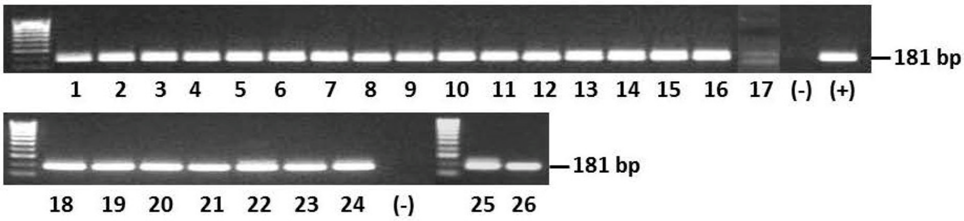

Figure S4. Nested PCR analysis of the Tax 181 pb amplimer in the 26 HTLV-1 positive LBC's. Primers used were 5′-TATCGGCTCAGCTCTACAGT-3′ and 5′-GGTGGGTTCCATGTATCCAT-3′. Conditions were 94 °C × 5 min and then 25 cycles of 94 °C × 15 s, 55 °C × 15 s, 72 °C × 15 s, 72 °C × 7 min.
